# Steroidogenic and maturation-inducing potency of native gonadotropic hormones in female chub mackerel, *Scomber japonicus*

**DOI:** 10.1186/1477-7827-10-71

**Published:** 2012-09-05

**Authors:** Hirofumi Ohga, Kensuke Kaneko, Akio Shimizu, Hajime Kitano, Sethu Selvaraj, Mitsuo Nyuji, Hayato Adachi, Akihiko Yamaguchi, Michiya Matsuyama

**Affiliations:** 1 Laboratory of Marine Biology, Faculty of Agriculture, Kyushu University, Fukuoka 812-8581, Japan; 2 National Research Institute of Fisheries Science, Fisheries Research Agency, Kanazawa, Yokohama 236-8648, Japan

**Keywords:** Chub mackerel, Perciform, Asynchronous, FSH, LH, Steroidogenesis, Oocyte maturation

## Abstract

**Background:**

The gonadotropins (GtHs), follicle-stimulating hormone (FSH) and luteinizing hormone (LH) are produced in the pituitary gland and regulates gametogenesis through production of gonadal steroids. However, respective roles of two GtHs in the teleosts are still incompletely characterized due to technical difficulties in the purification of native GtHs.

**Methods:**

Native FSH and LH were purified from the pituitaries of adult chub mackerel, *Scomber japonicus* by anion-exchange chromatography and immunoblotting using specific antisera. The steroidogenic potency of the intact chub mackerel FSH (cmFSH) and LH (cmLH) were evaluated in mid- and late-vitellogenic stage follicles by measuring the level of gonadal steroids, estradiol-17beta (Ε2) and 17,20beta-dihydroxy-4-pregnen-3-one (17,20beta-P). In addition, we evaluated the maturation-inducing potency of the GtHs on same stage follicles.

**Results:**

Both cmFSH and cmLH significantly stimulated E2 production in mid-vitellogenic stage follicles. In contrast, only LH significantly stimulated the production of 17,20beta-P in late-vitellogenic stage follicles. Similarly, cmLH induced final oocyte maturation (FOM) in late-vitellogenic stage follicles.

**Conclusions:**

Present results indicate that both FSH and LH may regulate vitellogenic processes, whereas only LH initiates FOM in chub mackerel.

## Background

In teleosts and other vertebrates, reproductive processes are regulated by a network of endocrine hormones on the brain–pituitary–gonad (BPG) axis. Pituitary gonadotropins (GtHs), follicle-stimulating hormone (FSH), and luteinizing hormone (LH), are key central signaling molecules on the BPG axis. GtHs regulate different stages of ovarian development by stimulating gonadal steroid production in the somatic cells surrounding the germ cells [1,2]. In mammalian vertebrates, the physiological roles of GtHs are well established; FSH regulates ovarian follicular development and LH promotes follicular maturation [3]. However, the role of GtHs in teleosts reproduction is still incompletely characterized, mainly due to diverse reproductive strategies and technical difficulties in the purification of native GtHs.

Specific roles for teleost GtHs have been revealed mainly in salmonids, which exhibit synchronous or group-synchronous ovarian development and spawn single batch of eggs. These features enable an easy correlation between changes in endocrine hormones and ovarian development. Moreover, homologous immunoassays for measuring pituitary/plasma FSH and LH and purified native GtHs are already available for salmonids. Plasma FSH levels are high during the early phase of vitellogenesis, whereas LH increases during the maturational phase such as final oocyte maturation (FOM) and ovulation in salmonids [4-7]. In addition, *in vitro* and *in vivo* experiments using purified GtHs have shown that FSH is involved in the vitellogenic growth of oocytes by stimulating estradiol-17β (Ε2) production, whereas LH mediates FOM through the production of the maturation-inducing steroid (MIS), 17,20β-dihydroxy-4-pregnen-3-one (17,20β-P) [8,9]. In contrast, species with asynchronous ovarian development show multiple batch spawning and exhibit complex dynamics in their follicular development. Since vitellogenesis and oocyte maturation occur simultaneously in one ovary [10], ovarian follicles are likely to be exposed to both FSH and LH regardless of their developmental status [11]. Hence, regulating the synthesis and secretion of pituitary GtH is likely more complicated during the spawning cycle.

Pituitary GtHs have been isolated in several fish species to clarify their roles in ovarian growth and maturation. Some *in vitro* experiments using purified GtHs have been performed in perciform fish (red seabream *Pagrus major*[12-14]; bigeye tuna *Thunnus obesus*[15]; European sea bass *Dicentrarchus labrax*[16]). In red seabream, LH stimulated E2 production by vitellogenic follicles in a dose-dependent manner; however, FSH showed no potency to stimulate E2 production [14]. In bigeye tuna, both FSH and LH stimulated E2 production by vitellogenic ovarian tissue [15]. In female European sea bass, FSH stimulated release of E2 from ovarian fragment in dose-dependent manner [16]. These results suggest that GtH functions differ depending on species in even the perciform fishes. Furthermore, no *in vitro* experiments on MIS production using purified GtHs have been performed in perciform fish.

The Japanese chub mackerel, *Scomber japonicus*, belongs to the order Perciformes, and is widely distributed throughout temperate and subtropical waters of the Pacific Ocean. This species is one of the most important commercially utilized fish in Japan. Similar to many other perciform fish, chub mackerel exhibits multiple spawning and asynchronous ovarian development [17]. Due to unreliable and unpredictable wild catches, aquaculture of the chub mackerel has commenced in southwestern Japan using young or adult fish captured from the wild. This system allows for fish sampling throughout the year to conduct endocrinological studies. Our group has already characterized the upstream signaling molecules to GtHs, namely kisspeptins and gonadotropin-releasing hormones, and has demonstrated their involvement in the reproductive cycle of chub mackerel [18-21]. Moreover, immunoreactive changes in pituitary FSH and LH content and transcriptional changes in pituitary GtH subunits during the seasonal reproductive and spawning cycles of female chub mackerel indicated that FSH is involved in vitellogenesis, whereas LH functions during both vitellogenesis and FOM [22,23].

In the present study, we purified chub mackerel FSH (cmFSH) and LH (cmLH) from the pituitaries of adult fish and analyzed their *in vitro* steroidogenic and maturation-inducing potencies to clarify the roles of FSH and LH during ovarian growth and maturation in chub mackerel.

## Methods

### Pituitary collection

Sexually mature male and female chub mackerel were obtained from Nagasaki Fish Market in March 2009, just prior to the spawning season. Whole pituitary glands (n = 90) were taken from both sexes and frozen immediately in liquid nitrogen and stored at −80°C until use.

### GtHs extraction

Pooled pituitaries were homogenized in 35% ethanol-10% ammonium acetate (pH 6.1) containing protease inhibitor cocktail (Complete; Boehringer Mannheim Gmbh Biochemica, Basel) on ice. The homogenate was placed for 18 h at 4°C and centrifuged to obtain supernatant. The cold ethanol corresponding to 4 times volume of supernatant was added slowly and kept at 4°C for 24 h. Ethanol-added supernatant was re-centrifuged for gaining precipitate.

### Chromatography procedure

The precipitate was dissolved in 20 mM ammonium bicarbonate (pH 8.7) and applied to a DEAE cellulose anion exchange chromatography (DEAE MemSEq 1010 cartridge, Millipore, MA) on anion-exchange high-performance liquid chromatography system. On this step, column was also equilibrated with 20 mM ammonium bicarbonate (pH 8.7). Adsorbed proteins were eluted with a linear gradient of 20–500 mM ammonium bicarbonate (pH 8.7) and fraction size was set to be 1 ml/tube. The part of fractions were lyophilized and subjected to an immunoblotting step.

### Electrophoresis

For biochemical analysis of purified proteins, 14% slab type of sodium dodecyl sulfate polyacrylamide gel electrophoresis (SDS-PAGE) and 7.5% slab type of native polyacrylamide gel electrophoresis (native-PAGE) in Tris-buffer system was operated. SDS-PAGE and native-PAGE procedures were carried out according to Laemmli [24]. After electrophoresis, both PAGE gels were stained with Coomassie brilliant blue (CBB).

### Western blotting

Western blotting procedure was carried out according to Shimizu and Yamashita [25]. Antisera used were Fh FSHβ 50–60 (003 antisera) and Fh LHβ 91–106 (299 antisera), raised against synthetic fragment peptides of mummichog *Fundulus heteroclitus* FSHβ and LHβ [25]. We already ascertained that both 003 and 299 antisera show strong immunoreaction for chub mackerel FSH and LH cells, respectively [22,26]. Subsequent to identification of the cmFSH and cmLH rich fractions using the above two antibodies, pooled fractions were concentrated using ultrafilter and tested directly for *in vitro* bioassay.

### N-terminal amino acid sequencing

To identify the GPα subunits, N-terminal amino acid sequencing was conducted. Protein samples were spotted onto the PVDF membrane and were subjected to N-terminal amino acid sequence analysis by a gas-phase protein sequencer (Applied Biosystems).

### Animals

Adult chub mackerel reared in sea pens at a fish farm were transported to the Fishery Research Laboratory, Kyushu University, Fukuoka Prefecture, and stocked in a concrete outdoor tank with running sea water. Captive female fish in this season has fully grown ovaries just prior to FOM [17]. Fish were killed by decapitation just before the assay and vitellogenic ovaries were removed and placed in ice-cold saline solution (0.8% NaCl containing 0.042% KCL, 0.025% CaCl_2_ and 0.02% MgCl_2_·6H_2_O). At the time of sampling, the fish were carefully treated and sacrificed following the guidelines for animal experiments in the Faculty of Agriculture and Graduate Course of Kyushu University, and in agreement with the laws (No. 105) and declaration (No. 6) of the Japanese Government.

### Steroidogenic potency and steroid analysis

Ovarian follicles were separated via gentle pipetting from the ovary in saline solution. To isolate individual follicles according to the follicular diameters, follicles were sorted using stainless mesh filters (Nippon Rikagaku Kikai, Tokyo) with apertures of 600 μm (for late-vitellogenic stage follicles; LV) and 355 μm (for mid-vitellogenic stage follicles; MV). Each stage follicles (100–130 follicles/well) were pre-incubated for 90 min in 24-well plates containing 1.0 ml hormone free Leibovitz’s L-15 culture medium (Sigma, St. Louis, Mo) (pH7.5) containing 10 mM HEPES and 0.02% Gentamycin sulfate (Sigma, St. Louis, Mo) in a temperature-controlled incubator at 18°C with shaking at 50 rpm. Subsequently, follicles were incubated in L-15 medium in the absence or presence of different concentrations of cmFSH or cmLH (6 to 200 ng/ml). The above concentrations were selected based on previous reports demonstrating production of sex steroids by native GtHs [8,14,15,27-31]. To examine the activity of cmFSH and cmLH relative to another gonadotropin, human chorionic gonadotropin (hCG; Aska Pharmaceutical, Tokyo, Japan) was tested at concentrations of 0.1 and 10 IU/ml. In addition, to examine the viability of isolated follicles, testosterone (Sigma, St. Louis, Mo) which is a precursor of E2 was incubated with the follicles and E2 content produced was measured. All treatments were analyzed in two replicates. After incubation at 18°C for 18 h with shaking at 50 rpm, media were collected and stored at −20°C until use. Each treatment was repeated representing three different ovaries. E2 and 17,20β-P levels in incubation medium were measured using an Estradiol EIA Kit (Cayman, MI) and ELISA according to Matsuyama et al. [32], respectively.

### Maturation-inducing potency

Ovaries, same of steroidogenic experiment, were minced into small tissue fragments (about 20 mg) in saline solution. Each fragment was pre-incubated for 90 min in 6-well plates containing 3.0 ml hormone-free L-15 culture medium at 18°C with 50 rpm shaking. Subsequently, ovarian fragments were incubated in L-15 medium in the absence or presence of different concentrations of cmFSH and cmLH (6 to 200 ng/ml) for 24 h at 18°C with 50 rpm shaking. After incubation, fragments were fixed in clearing solution (ethanol: formalin: acetic acid = 6:3:1), and individual follicles were isolated by pipetting, and subjected to stereomicroscopy. FOM was confirmed by the germinal vesicle migration and % maturation was calculated as maturation follicles/LV stage follicles. The experiment was repeated three individual ovaries.

### Statistical analysis

Data were expressed as means ± SEM (standard errors of the mean), and analyzed by one-way ANOVA followed by a Bonferroni's Multiple Comparison Test using Prism 4 (GraphPad Software, San Diego, CA).

## Results

### Purification and biochemical properties of cmFSH and cmLH

**Figure 1:**
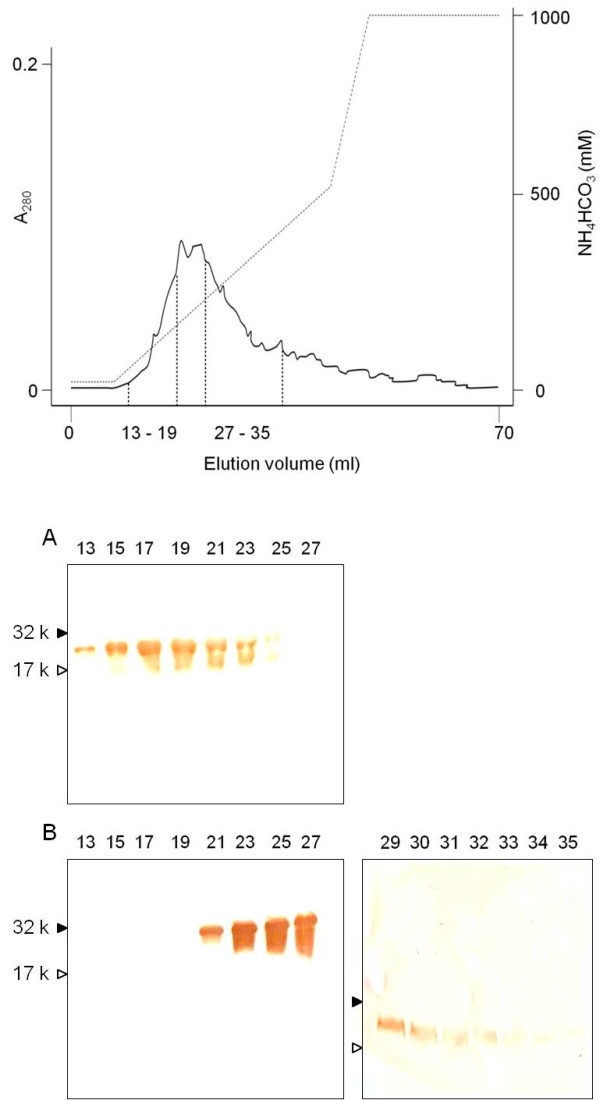
**Anion-exchange chromatography of the ethanol extract and western blotting of eluted fractions. (*Upper*)** Absorbed proteins were eluted with a liner gradient of 20–500 mM ammonium bicarbonate (pH 8.7) from chub mackerel pituitaries on DEAE MemSEq 1010 cartridge. Fractions absorbance read at 280 nm. **(*Lower*)** (**A**) Western blotting with 003 antisera. (**B**) Western blotting with 299 antisera. Lane number indicates fraction number. Antisera used were Fh FSHβ 50–60 (003 antisera) and Fh LHβ 91–106 (299 antisera), raised against synthetic fragment peptides of mummichog (*Fundulus heteroclitus*) FSHβ and LHβ [25].

**Figure 2:**
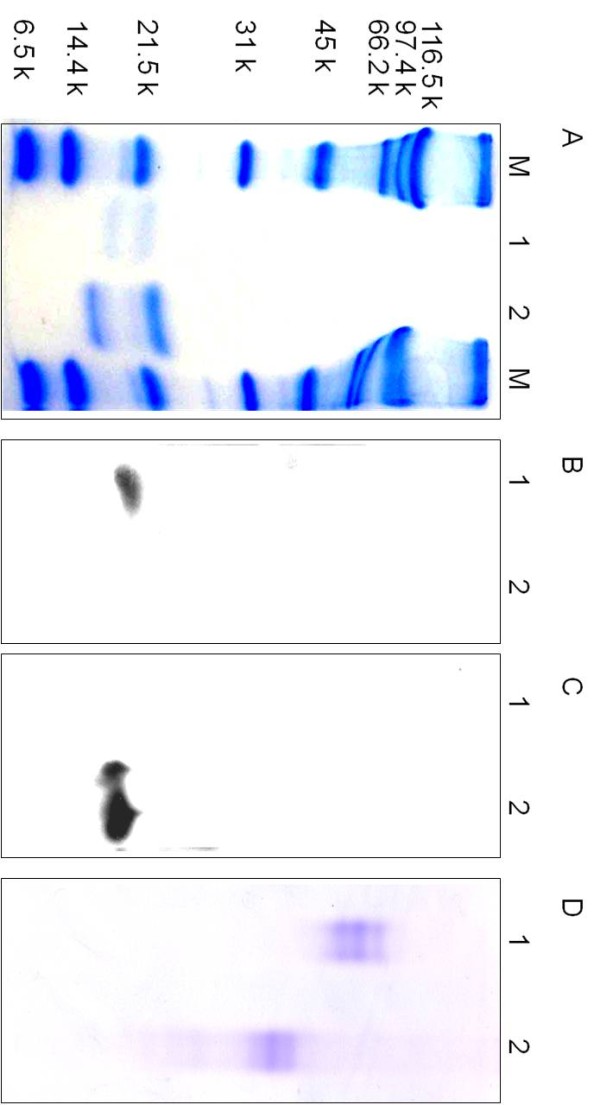
**SDS-PAGE, Western blotting and native-PAGE analysis of purified cmFSH and cmLH. (A**) 14% SDS-PAGE. Proteins reduced with 2-mercaptethanol and stained with Coomassie brilliant blue. (**B**) Western blotting with 003 antisera. (**C**) Western blotting with 299 antisera. (**D**) 7.5% native-PAGE. Proteins stained with Coomassie brilliant blue. Lane 1: A sample from the pooled fractions 13–19 in Figure 2 for cmFSH; Lane 2: A sample from the pooled fractions 20–26 in Figure 2 for cmLH.

Ethanol-extracted glycoproteins from chub mackerel pituitaries were separated by DEAE anion-exchange chromatography (Figure 1*Upper*). The results of Western blotting revealed that fractions only immunoreacted to the 003 antisera (fractions 13–19, Figure 1*Lower* A) and were pooled and preserved as native FSH. In fraction 29–35, no immunoreaction was observed to the 003 antisera (Data not shown). Moreover, fractions that only immunoreacted to the 299 antisera (fraction 27–35, Figure 1*Lower* B) were pooled and preserved as native LH. Fractions 20–26 contained both FSH and LH (Figure 1*Lower*). CBB staining under reducing conditions (2ME+) revealed that the expected molecular weight of the cmFSH α and β subunits was approximately 22 and 18 kD, and that the cmLH α and β subunits were approximately 23 and 15 kD, respectively (Figure 2A). In the Western blot analysis, the purified cmFSHβ and cmLHβ subunits reacted specifically to the 003 and 299 antisera, respectively (Figure 2B and 2C). The α subunits of cmGtHs showed N-terminal amino acid sequences (PNVD) which corresponded to those deduced from chub mackerel GPα cDNA data [23]. In the native-PAGE, cohesive 3 bands were observed for both cmFSH and cmLH and the other bands were not ascertained (Figure 2D). The cohesive 3 bands suggest differences with the degree of glycosylation. The mobility between cmFSH and cmLH was different and this might be caused by differences with molecular size between cmFSH and cmLH.

### Gonadal steroid production

**Figure 3:**
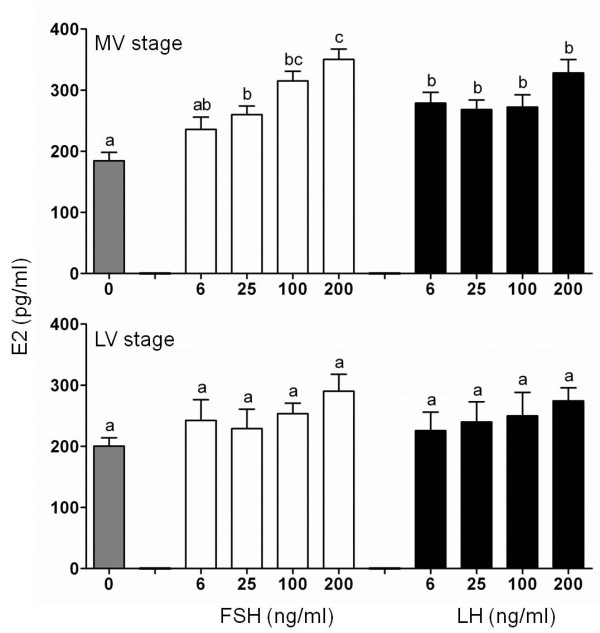
***In vitro *ovarian E2 production.***In vitro *effects of purified cmFSH and cmLH on E2 production by chub mackerel mid-vitellogenic (MV) and late-vitellogenic (LV) stage ovarian follicles. Values are expressed as the mean ± SEM. Different letters above the bars represent significant differences (P < 0.05) between hormone doses.

**Figure 4:**
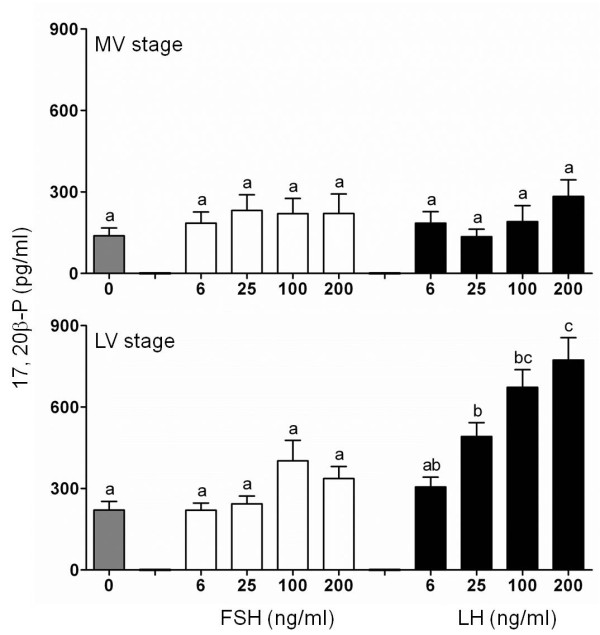
***In vitro *ovarian 17,20β-P production.***In vitro *effects of purified cmFSH and cmLH on 17,20β-P production by chub mackerel mid-vitellogenic (MV) and late-vitellogenic (LV) stage ovarian follicles. Values are expressed as the mean ± SEM. Different letters above the bars represent significant differences (P < 0.05) between hormone doses.

The steroidogenic potencies of cmFSH and cmLH were evaluated using actively vitellogenic (mid-vitellogenic, MV) and fully grown (late-vitellogenic, LV) follicles. E2 production was significantly induced by cmFSH in a dose-dependent manner in MV stage follicles but not in LV follicles (Figure 3). At all dose tested, cmLH significantly stimulated E2 levels in MV stage follicles without any differences between doses (Figure 3). Only cmLH stimulated 17,20β-P production in a dose-dependent manner in LV follicles (Figure 4). However, 17,20β-P was not produced in response to cmLH in MV follicles.

### Bioactivity of cmFSH and cmLH in relative to another gonadotropin

**Figure 5:**
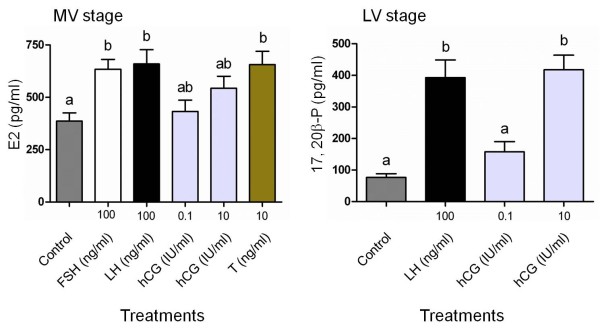
**Bioactivity of cmFSH and cmLH relative to another compound. **Steroidogenic potency of native FSH and LH relative to hCG or testosterone in chub mackerel mid-vitellogenic (MV) and late-vitellogenic (LV) stage ovarian follicles. Values are expressed as the mean ± SEM. Different letters above the bars represent significant differences (P < 0.05) between hormone doses.

E2 concentrations in media from MV follicle incubations were elevated in the presence of cmFSH, cmLH and testosterone, while no significant difference was found with hCG (Figure 5). In the LV stage follicles, cmLH and 10 IU/ml of hCG stimulated 17,20β-P production; no significant difference was detected between their productions (Figure 5).

### Maturation-inducing potency

**Figure 6:**
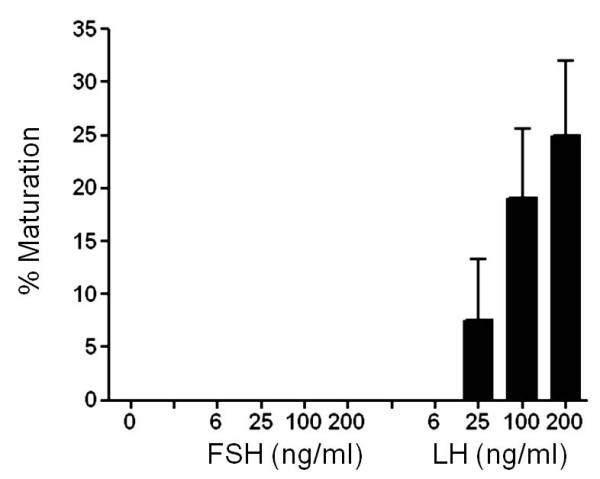
**Probability of final oocyte maturation *in vitro*. ***In vitro *effects of purified cmFSH and cmLH on the percentage of maturation inducing by chub mackerel ovarian fragments. Tissue fragments (20 mg/well) were incubated with 3 ml of control medium or medium containing different concentrations of cmGtHs. Values are expressed as mean ± SEM.

Only cmLH increased the percentage of maturing oocytes in a dose-dependent manner (Figure 6).

## Discussion

We purified native GtHs from chub mackerel pituitaries and conducted a functional evaluation using intact ovarian follicles. First, we purified and isolated native FSH and LH from chub mackerel pituitaries using anion-exchange chromatography and an immunochemical method. The purified cmFSH and cmLH strongly reacted with the 003 and 299 antisera, respectively. Most previous studies on the purification of native FSH and LH in fish used stepwise chromatography to screen protein fraction and evaluated each fraction by physicochemical assays such as SDS-PAGE [15,28,29,31,33-35]. However, the immunochemical method adopted in the present study has advantages over a preceding method, as it can save stepwise chromatography process time and sample quantity [25]. Separation of intact FSH and LH from native mixture is generally difficult in fishes. Their physiochemical properties resemble each other, and diversities in the carbohydrate chains inhibit precise separation using various chromatography procedures. Reverse-phased high-performance liquid chromatography under neutral conditions has been used for several fish species, such as skipjack tuna *Katsuwonus pelamis*[34] and the Mediterranean yellowtail *Seriola dumerilii*[35]. However, this method has a disadvantage in that LH molecules may dissociate into subunits in some fish preparations. Such cases are observed in bigeye tuna [15], mummichog (unpublished results), and chub mackerel (unpublished results). Hydrophobic chromatography has been used to separate FSH and LH in the mummichog [25], but this method has another disadvantage in that yields are considerably low probably because of adsorption. The method used in the present study (DEAE anion exchange chromatography with ionic strength gradient increase in the mobile phase) has no such disadvantages, although complete separation of FSH and LH is impossible. This method yielded a considerable amount of an FSH and LH mixture (Figure 1*Upper*), which is difficult to purify further. However, combined with Western blot analysis using highly specific antibodies, contaminants could be completely excluded from both the FSH and the LH fractions (Figure 1*Lower*). Therefore, the procedures described here may be convenient methods for obtaining native GtHs from various fish species, when a pair of highly specific anti-FSH and anti-LH antibodies is available.

The results of a functional evaluation indicated that both cmFSH and cmLH are capable of stimulating E2 production in MV stage follicles (Figure 3). Further, incubation of MV stage follicles in the presence of gonadotropin, hCG (0.1-10 IU/ml) or an aromatizable androgen, testosterone (T, 10 ng/ml) resulted in the E2 production, with the latter showing a significantly higher levels on par with cmFSH and cmLH (Figure 5). In the vitellogenic follicles of salmonids, GtH has been shown to stimulate T production in the theca cells, which diffuses into granulosa cells and aromatized to E2 [36,37]. In the vitellogenic follicles of chub mackerel, Ε2 is synthesized from pregnenolone via T [38]. Results obtained indicates that not only isolated MV stage follicles are viable with aromatase (cytochrome P450 aromatase [39]) activity to convert T into E2 but also purified cmFSH and cmLH have enough biological potency.

The *in vitro* effects of purified GtHs on E2 production have been demonstrated in several perciform fish. FSH significantly stimulates the release of E2 in early and mid vitellogenic European sea bass ovarian fragments [16]. Similarly, both FSH and LH stimulate E2 production in bigeye tuna; however, LH is more potent than FSH [15]. In contrast, the biological activity of FSH is lower than that of LH for inducing *in vitro* E2 production by vitellogenic ovarian fragments in red seabream [14]. As E2 is synthesized by ovarian follicles surrounding oocytes, the differences in FSH or LH activity on follicular steroidogenesis may differ depending on whether ovarian fragments or different stages of vitellogenic follicles are used in culture. The present study clearly found that cmFSH or cmLH stimulated E2 production in MV stage follicles, but not LV follicles. Moreover, these different results on the effect of GtHs on E2 production could be attributed to species-specific factors in multiple-spawning species. In MV stage follicles, E2 production by cmLH reached a plateau between 6 and 200 ng/ml. Likewise in bigeye tuna, E2 production in vitellogenic ovarian fragments by FSH increased in a dose-dependent manner, while LH reached plateau faster than FSH [15]. This result shows similarity with our present data. However, at present stage, physiological difference between FSH and LH in the steroidogenic potency remains unclear.

Several *in vitro* studies have reported that both FSH and LH stimulate E2 production in vitellogenic follicles (salmonids [8,27], common carp *Cyprinus carpio*[29], and bigeye tuna [15]) like in chub mackerel. Further, in salmonids [40,41] and catfish *Clarias gariepinus*[42,43], it has been indicated that both FSH and LH bind to the FSHR with similar affinities. Pituitary FSHβ and LHβ immunoreactive levels in chub mackerel suggest that both FSH and LH involve in vitellogenesis [22]. Taking above into consideration, it is possible that the E2 produced by cmLH might be due to cross-activation between cmLH and FSHR. Future studies on the expression dynamics of their cognate receptors by vitellogenic follicles, reporter-gene assays of cognate ligand-receptor interactions, and circulating levels of FSH and LH during vitellogenesis will help to clarify this possibility.

cmLH but not cmFSH was capable of stimulating 17,20β-P production in LV stage follicles (Figure 4) including the potential to induce germinal vesicle migration *in vitro* (Figure 6). Further, production levels of 17,20β-P by hCG in LV stage follicles resembled those of cmLH, showing that cmLH had comparable potency to 10 IU/ml of hCG in stimulating 17,20β-P production (Figure 5). After vitellogenesis, the steroidogenic pathway shifts from E2 to 17,20β-P [37], and 17,20β-P is highly effective at inducing FOM *in vitro* and acting as a MIS in chub mackerel [38]. Our recent study indicated that LHβ-immunoreactive and LHβ mRNA levels were much higher in the pituitary of spawning chub mackerel [22,23] than in fish of other maturational stages. The results of the present study indicate that LH is responsible for FOM in chub mackerel. The maturation-inducing potency of purified FSH and LH demonstrated in this study are in agreement with other reports on salmonids and red seabream [8,13,30].

## Conclusions

In the present study, a highly active purified preparation of FSH and LH was obtained from chub mackerel pituitaries, and analyses suggested that cmFSH and cmLH are capable of stimulating E2 production by MV stage follicles. Furthermore, cmLH showed higher potency than cmFSH for stimulating 17,20β-P production and inducing FOM in LV follicles.

## Competing interests

The authors declare that they have no competing interests.

## Authors contributions

HO carried out the *in vitro* bioassay, data analysis and interpretation, and drafted the manuscript. KK participated in the preliminary *in vitro* bioassay. AS carried out the hormonal purification and helped to draft the manuscript. KH, SS, MN and HA assisted the experiments. AY assisted with data interpretation. MM participated in study design, assisted with data interpretation and supervised this work. All authors read and approved the final manuscript.
